# Darwin's Manufactory Hypothesis Is Confirmed and Predicts the Extinction Risk of Extant Birds

**DOI:** 10.1371/journal.pone.0005460

**Published:** 2009-05-06

**Authors:** David G. Haskell, Anupam Adhikari

**Affiliations:** Department of Biology, University of the South, Sewanee, Tennessee, United States of America; McGill University, Canada

## Abstract

In the Origin of Species Darwin hypothesized that the “manufactory” of species operates at different rates in different lineages and that the richness of taxonomic units is autocorrelated across levels of the taxonomic hierarchy. We confirm the manufactory hypothesis using a database of all the world's extant avian subspecies, species and genera. The hypothesis is confirmed both in correlations across all genera and in paired comparisons controlling for phylogeny. We also find that the modern risk of extinction, as measured by “Red List” classifications, differs across the different categories of genera identified by Darwin. Specifically, species in “manufactory” genera are less likely to be threatened, endangered or recently extinct than are “weak manufactory” genera. Therefore, although Darwin used his hypothesis to investigate past evolutionary processes, we find that the hypothesis also foreshadows future changes to the evolutionary tree.

## Introduction

In the second chapter of the Origin of Species Darwin hypothesized that the “manufactory” of species operates at different rates in different lineages, and that taxonomic richness is therefore autocorrelated between ancestral and descendent branches within the evolutionary tree [1]. He predicted that speciose genera should be comprised of species made up of many “varieties or incipient species”. His hypothesis focused on genera, species and subspecies – the “tips” of the evolutionary tree.

Darwin tested his prediction with lists of English plants (from twelve counties) and beetles (from two districts). He divided his list into two “sides” and found, as he predicted, “that a larger proportion of the species on the side of the larger genera present varieties, than on the side of the smaller genera”. Darwin then used this data as evidence that rates of cladogenesis are autocorrelated between ancestral and descendent branches within the evolutionary tree (and, by extension, he used his data as evidence against special creation). Darwin's manufactory hypothesis therefore uses the shape of the evolutionary tree (inferred by Darwin from the richness of taxonomic hierarchies) to test hypotheses about the process of evolution.

Although there have been several modern investigations of cladogenesis and tree shape [2], [3], the manufactory hypothesis has not, to our knowledge, been tested since Darwin. Darwin's formulation of the manufactory hypothesis examines autocorrelation of richness between ancestral and descendent branches within the evolutionary tree, and therefore provides a complement to studies of tree topology, shape and balance [2], [4]. This paper follows Darwin by examining autocorrelation among ancestral and descendent branches of the tree at the level of subspecies through genera and we also extend the comparison to the level of families. Lineages also extend back to more ancient parts of the evolutionary tree, but here we do not examine these older patterns.

The taxonomic hierarchy reflects past patterns of diversification. The current extinction crisis may rework macroevolutionary patterns of biodiversity, affecting the future shape of the evolutionary tree of life [5]. Darwin's manufactory hypothesis can therefore be extended to examine the link between past and future patterns of diversity. If genera that have been “strong manufactories” in the past are less likely than average to be endangered in the present, then there appears to be a connection between past and present processes. If such genera are equally or more likely than average to be endangered, then past diversification may be de-coupled from present extinction risk.

Here, we use a database of all the world's avian subspecies, species and genera to test Darwin's manufactory hypothesis. Specifically, we test the prediction that speciose genera should be comprised of species containing many subspecies. We test these predictions by running correlations across all of the world's avifauna and with paired comparisons controlling for phylogeny. We also test the hypothesis that the extinction risk of extant bird species is associated with the species' position relative to Darwin's manufactory schema. Our analysis, like Darwin's, uses the taxonomic hierarchy as a surrogate for the shape of the evolutionary tree. There is currently no complete phylogeny of birds to the subspecific level, so our analysis is provisional and depends on the assumption that taxonomic hierarchies contain information about the branching patterns of evolutionary trees. We assess the validity and limitations of this assumption in the Discussion.

## Methods

### Analysis across all genera with no phylogenetic correction

The number of species per genus was tallied using Dickinson's [6] database of the world's birds. For each species (n = 9723) within all genera (n = 2161) , the number of subspecies (n = 26,416) was tallied, then the mean number of subspecies was computed for each genus. Thus, each genus was characterized by two numbers: the number of species the genus contains and the mean number of subspecies per species.

We used non-parametric Kendall correlations to test for an association between the speciosity of genera and the mean and median number of subspecies per species. In addition, although Darwin did not extend his hypothesis above the level of genera, we added an analysis to test for a correlation between the number of genera per family and the mean number of species per genus (in effect, examining one level further up the taxonomic hierarchy than Darwin's original analysis). This analysis also used a non-parametric Kendall correlation.

In addition, for each family that contained at least three genera (n = 98 families out of a total of 194), we fitted a least squares line through the number of subspecies per species plotted against the number of species per genus. We analyzed these data with a one-tailed Binomial test with the null hypothesis that an equal number of least squares slopes should be positive and negative.

### Analysis across a sub-sample of genera, controlling for phylogeny

Darwin's hypothesis predicts that for closely related genera (e.g., sister genera), the genus in each pair that has the highest number of species should also have the highest mean number of subspecies per species on average. To test this prediction we used pairs of genera from previously published phylogenetic hypotheses [7]–[14]. We used pairs of genera that were reciprocally monophyletic in the published trees (Table S1). Within the paired genera we used a one-tailed binomial test whether the genus with the highest number of species also had the highest number of subspecies per species.

### Conservation Status Analysis

To examine whether or not an association exists between the conservation status of species within genera and their position in Darwin's manufactory schema, all the world's bird genera were divided into four groups based on their speciosity and the richness of their subspecies. We used the median richness of species per genus (median = 2 species per genus) and subspecies per species (median = 2 subspecies per species) as dividing lines, generating the following categories of genera: those with high richness of species and high richness of subspecies, those with low richness of species and low richness of subspecies, those with high richness of species and low richness of subspecies, and those with low richness of species and high richness of subspecies.

Under the null hypothesis of random distribution of threatened species among genera, the expected number of threatened species in each genus is the number of species in the genus multiplied by the proportion of all species that are threatened. When calculated across genera, the difference between the actual number of threatened species per genus and the expected number of threatened species per genus would, under the null hypothesis, have a mean of zero. If the conservation status of genera is unrelated to Darwin's schema, then all categories of genera should conform to this null hypothesis. If, however, some categories of genera are more or less likely to be threatened than would be expected by chance, then the mean of the differences between the actual number of threatened species per genus and the expected number of threatened species per genus for these categories would be greater or less than zero.

We used two measures of conservation status. First, for each genus, we tallied the number of species in the World Conservation Union (WCN) “Red List” database of Extinct, Extinct in the Wild, Critically Endangered, Endangered, Vulnerable, and Near Threatened species [15]. This includes all species that the WCN considers to be of conservation concern. Second, we tallied only those species in the Extinct, Extinct in the Wild or Critically Endangered groups. This includes only those species that are extinct or very close to extinction. The actual and expected numbers of species of conservation concern were compared across the four categories of genera (high/high, low/low, high/low, low/high) using non-parametric tests to compare groups. We used multi-response permutation procedures that compare Euclidean distances among groups of numbers (Blossom, Version W2007.12.21). This test is analogous to a one-way analysis of variance, but makes no assumptions about the distribution of the data [16], [17].

## Results

**Figure 1:**
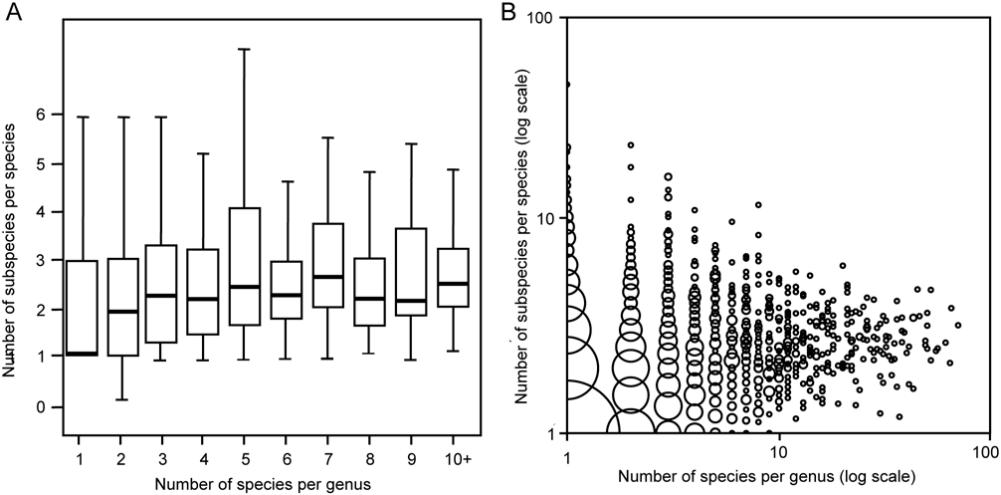
More speciose genera tend to be comprised of species with many subspecies. The graph summarizes data from all the world's bird genera, species and subspecies. A: boxplot shows medians (bold lines), quartiles (boxes), and largest and smallest observed values that are less than the 1.5 times the interquartile range (whiskers). B: scattergraph of the same data plotted on log axes with the total area of each point proportional to the number of genera at that point. For points that are bisected by one or both of the axes, the area of each point that represents the sample size at that point is the area of the complete point circle, even if only part of the point circle is shown.

Across all genera, speciose genera tended to contain species that had higher mean numbers of subspecies (Figure 1; Kendall Correlation Tau = 0.23, p<0.0001). In addition, families with many genera tended to be comprised of genera with a high mean number of species (Kendall Correlation Tau = 0.22, p<0.0001). These correlations also held if median numbers of subspecies and species were used instead of means (in both cases p<0.0001).

Within the 98 families with at least three genera, 59 families had a positive relationship between the speciosity of genera and the mean number of subspecies per species within those genera (one-tailed Binomial test, p = 0.027). The remaining families had negative relationships.

There were 46 pairs of genera in the independent pairs analysis. Twenty one pairs had the same number of species in each; of the 25 remaining pairs, 20 genera with the higher number of species also had the highest mean number of subspecies per species (one-tailed Binomial test, p = 0.002).

**Figure 2:**
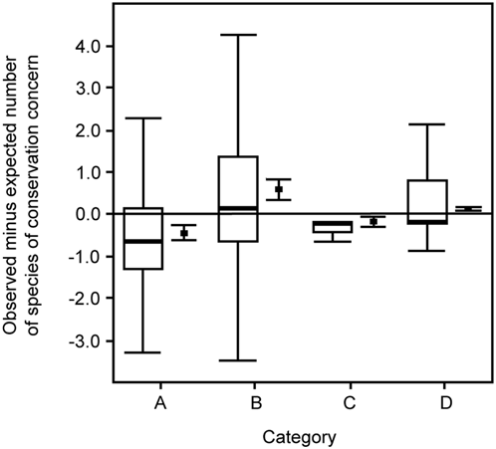
Darwin's categories of genera differ in the relative number of species of conservation concern. Open bars (leftmost bar for each category) show medians (bold lines), quartiles (boxes), and largest and smallest observed values that are less than the 1.5 times the interquartile range (whiskers). Closed points and whiskers (rightmost bar for each category) show means and 95% confidence intervals. Categories are: A, species-rich genera with many subspecies (n = 547); B, species-rich genera with few subspecies (n = 328); C, species-poor genera with many subspecies (n = 392); D, species-poor genera with few subspecies (n = 894). Means and medians below the horizontal line have fewer species of conservation concern than would be expected by chance; means and medians above this line have more species of conservation concern than would be expected by chance.

The categories of genera differed in their relative degrees of conservation concern as described by WCN (Figure 2). This result held both when all species of conservation concern were analyzed (MRPP test statistic = −187.4; p<0.0001; n = 2127 species) and when only extinct or critically endangered species were analyzed (MRPP test statistic = −210.0; p<0.0001; n = 306 species). In pairwise comparisons, each category was significantly different from all other categories (MRPP test statistics<−36.6; p<0.0001). The Red List [15] and Dickinson [6] databases differ in some details of avian classification (lumping or splitting species within genera; 22% of genera had some degree of disagreement about the number of species in the genus; for those genera for which there was disagreement, the mean and median discrepancies between the number of species in the genus in the two databases were 2.44 and 1.00, respectively). Therefore, we also conducted the Red List analysis on the subset of the genera for which the two databases agree in every taxonomic detail and we found the same pattern (all Red List species: MRPP test statistic = −163.9; p<0.0001; n = 1176 species; and only extinct/critically endangered species: MRPP test statistic = −189.8; p<0.0001; n = 155 species).

## Discussion

We found that, as Darwin predicted, speciose genera of birds tend to be comprised of species that had many subspecies. In addition, this taxonomic autocorrelation in richness extends above the taxonomic levels examined by Darwin: we found that bird families within many genera tended to be comprised of genera with many species. There is substantial scatter around these trends, as indicated by their relatively low correlation coefficients (all coefficients were below 0.3; see also Figure 1). Darwin anticipated this variability, noting that rates of diversification were “generally…on an average” autocorrelated between ancestral and descendent branches that many exceptions to the trend were to be expected as the diversity of genera waxed and waned [1]. Despite this variability, we found that the trend was present at many levels of analysis: across all birds, within the majority of families, and in paired comparisons controlling for phylogeny.

Darwin assumed that the richness of the taxonomic hierarchy was correlated with the branchiness of evolutionary trees (“branchiness” refers to the extent of diversity within a part of the evolutionary tree; branchy parts of the tree are comprised of many lineages). Because we lack a complete phylogeny of birds to the sub-specific level, we cannot fully assess this assumption. The available data are ambiguous on this point – the relationship between taxonomies and evolutionary trees is controversial [18], [19]. In particular, the question of whether currently recognized avian subspecies match phylogenetic reality is unresolved. For example, Zink [20] claims that “a massive reorganization of classifications is required so that the lowest ranks, be they species or subspecies, reflect evolutionary diversity.” But Phillimore & Owens [21], in a later review of a larger sample of species, counter that “avian subspecies often provide an effective short-cut for estimating patterns of intraspecific genetic diversity, thereby providing a useful tool for the study of evolutionary divergence and conservation.”

Our analysis does not assume that every subspecies matches a monophyletic unit. Rather, we assume that, on average, those bird species with many subspecies have more phylogenetic structure within them than species with no or few subspecies. This assumption can be met without perfect concordance between subspecies taxonomy and true evolutionary trees. Phillimore & Owens' [21] analysis suggests that this phylogenetic signal may indeed be present, albeit imperfectly, in the taxonomic hierarchy. Another possibility is that subspecies richness reflects the extent of morphological diversification [22], [23], but not always overall phylogenetic structure. If this were the case, Darwin's manufactory hypothesis would inform us about autocorrelation in taxonomically important phenotypic characters, but not necessarily about true phylogenies. Likewise species and genera differ in their ages [24], [25], so taxonomic autocorrelation in patterns of diversity in species and genera does not necessarily imply autocorrelation in absolute rates of diversification, but may reflect autocorrelation in phenotypic characters used in taxonomic classifications.

Darwin thought that differences in diversity among taxa reflected different rates of origination (of species and subspecies). His choice of the term “manufactory” to describe these differences reflects that belief. But differences in diversity among extant taxa reflect not just rates of origination, but are also determined by rates of extinction [4]. Therefore, Darwin's preliminary analysis, and our confirmation of his predictions within birds, provide information about the pattern of evolution, but cannot tell us whether this pattern was caused by variation in speciation, extinction, or some combination of both. Thus, Darwin's metaphor should be extended to include the disappearance of the products of the manufactory: strong manufactories may have little to show if their wares vanish on the way to market. These “manufactories” are analogous to more recent metaphors such as “Adam and Eve” lineages – all are sources of ongoing cladogenesis [26].

Our finding that the richness of bird taxa is autocorrelated across the levels of the taxonomic hierarchy parallels the results of previous studies that have examined autocorrelation at genetic and ecological levels. Drummond et al.'s [27] study of rates of nucleotide substitution found weak or no autocorrelation in substitution among closely related lineages, but predicted much higher levels of autocorrelation among organisms that vary in their life-history or proof-reading mechanisms. Their study did not address autocorrelation in branchiness of trees. Ecological studies have likewise examined autocorrelation among levels of the taxonomic hierarchy [28]–[30]. In general, field surveys of diversity find that the richness of families or genera can serve as a good predictor of the richness of species, when samples are compared within a particular biome. The fact that higher taxonomic levels (e.g., genera) can serve as a surrogate for lower levels (e.g., species) may be due to the patterns of autocorrelation that we report here, but it also depends on how the various levels of diversity are spatially distributed across the landscape (e.g., if species are spatially clumped, whereas genera are not, we might expect a different correlation than if species were unclumped). These ecological studies are focused on developing effective field methods for monitoring biodiversity and they do not examine the taxonomic basis of the trends they report (as we do here), nor did they address the question of subspecies.

The confirmation of Darwin's hypothesis potentially informs the long-running debate about whether subspecies can be regarded as incipient species [20], [21], [23]. The positive autocorrelation between richness of species within genera and richness of subspecies within species suggests that bird subspecies are incipient species or, as discussed above, that there is autocorrelation among taxonomic levels in phenotypic characters that lead taxonomists to sub-divide taxa. Either way, the pattern that we describe here suggests that bird subspecies may be regarded as units in the process of either cladogenesis or of phenotypic divergence. If true, then as pointed out by Rabosky & Lovette [31], reports of declining rates of cladogenesis [32] may be an artifact of the omission of subspecies from the analyses. We emphasize that this application of our results to the debate about subspecies is speculative. The debate's resolution awaits finer-scaled information about the relationships among population structure, taxonomic hierarchies and evolutionary divergence.

Our results also suggest that current extinction events are not de-coupled from past evolutionary processes. Current extinction and near-extinction of species are known to be associated with ecological and life history variables such as body size, geographic range and fecundity [33]–[36]. We found that species loss or endangerment within birds is also correlated with the shape of the taxonomic hierarchy at the levels of genera through subspecies. Our results indicate that the current extinction crisis will have macroevolutionary consequences, trimming the avian tree of life in a way that over-represents some branching patterns relative to others, leaving the world disproportionately populated with genera from the “strong manufactory” category of Darwin's schema. This result extends Russell et al.'s [37] finding that extinction risk in extant birds and mammals is selective with respect to families and genera. Our results also concord with Sepkoski's [38] analysis of the marine fossil record that found that diversity and rates of origination at the level of orders was correlated with diversity and origination at the species level. Here we find that selectivity relates to not just overall taxonomic richness, but also to the degree of autocorrelation among taxonomic levels. Those clades that are rich at the levels of both genera and species (i.e., many species within genera and many subspecies within species) are significantly less likely to be threatened than are other clades (Figure 2). Our results apply within birds – whether these patterns are found in other taxa awaits further analysis.

Recently, conservationists have used information about the shape of evolutionary trees when prioritizing species of conservation concern. For example, the “evolutionarily distinct, globally endangered” program [39] rates species using both the status of their current populations and phylogenetic information about their “distinctiveness”. “Evolutionary distinctive” species are those at the end of bare branches on the evolutionary tree (few close relatives and long branch lengths; “weak manufactories” in Darwin's schema; Rodrigues et al. [40] review the related concept of “phylogenetic diversity” in conservation). Our analysis suggests that within birds these evolutionarily distinct species are more likely to be endangered than are species from very twiggy branches of the tree. Just as previous mass extinctions were selective and reworked macroevolutionary patterns in ways that differed from “background” times [41], it appears that the balance among the various categories of bird genera will shift during the current extinction event, leaving an evolutionary tree with proportionately more twiggy branches. This outcome is generally considered to be more harmful to conservation values than the alternative possibility of extinction focused on twiggy branches [40]; [but see 42]. If, as we find, patterns of current species extinctions have phylogenetic structure, then conservation efforts might more effectively protect biodiversity if they take this structure into account. Our results therefore provide support for recent efforts to incorporate phylogenetic information into conservation monitoring and planning [39], [43].

## Supporting Information

Table S1 Pairs of genera used in the analysis controlling for phylogeny. We used studies that sampled all, or nearly all, of the genera within the taxa of interest. Where more than one study sampled the same genera, we paired genera only if they appear as pairs in all the studies that included them in their sampling. Genera that were not recognized by Dickinson [6] were excluded. Taxon sampling in the studies used was as follows: Lovette & Bermingham [7]: “Representatives of all 25 extant genera currently placed in the Parulidae”. Lerner & Mindell [8]: “representatives of all 14 Accipitridae subfamilies, focusing on four subfamilies of eagles (booted eagles, sea eagles, harpy eagles, and snake eagles) and two subfamilies of Old World vultures (Gypaetinae and Aegypiinae) with nearly all known species represented.” Benz et al. [9]: “46 picid species, representing 24 of 28 currently recognized genera”. Baker et al. [10]: “90 out of 96 putative genera of Charadriiformes”. Griffiths et al. [11]: “we recognize 67 genera [of Accipitridae]… We sampled 54 of these genera.” Lovette & Rubenstein [12]: “at least one representative of all morphologically or biogeographically distinctive lineages in the Sturnidae and Mimidae”. Ohlson et al. [13]: “23 of the 24 genera of Cotingidae”. Lerner et al. [14]: “at least one individual of each nominal genus and species, and nearly all sub-species, of sub-buteos.”
(0.09 MB DOC)
