# Supplementary material for: Darwin's Manufactory Hypothesis Is Confirmed and Predicts the Extinction Risk of Extant Birds
Source: PLoS One. 2009 May 6;4(5):e5460. doi: 10.1371/journal.pone.0005460 (PMC2674221; doi:10.1371/journal.pone.0005460)
Supplement: Table S1 — Pairs of genera used in the analysis controlling for phylogeny. We used studies that sampled all, or nearly all, of the genera within the taxa of interest. Where more than one study sampled the same genera, we paired genera only if they appear as pairs in all the studies that included them in their sampling. Genera that were not recognized by Dickinson [6] were excluded. Taxon sampling in the studies used was as follows: Lovette & Bermingham [7]: “Representatives of all 25 extant genera currently placed in the Parulidae”. Lerner & Mindell [8]: “representatives of all 14 Accipitridae subfamilies, focusing on four subfamilies of eagles (booted eagles, sea eagles, harpy eagles, and snake eagles) and two subfamilies of Old World vultures (Gypaetinae and Aegypiinae) with nearly all known species represented.” Benz et al. [9]: “46 picid species, representing 24 of 28 currently recognized genera”. Baker et al. [10]: “90 out of 96 putative genera of Charadriiformes”. Griffiths et al. [11]: “we recognize 67 genera [of Accipitridae]… We sampled 54 of these genera.” Lovette & Rubenstein [12]: “at least one representative of all morphologically or biogeographically distinctive lineages in the Sturnidae and Mimidae”. Ohlson et al. [13]: “23 of the 24 genera of Cotingidae”. Lerner et al. [14]: “at least one individual of each nominal genus and species, and nearly all sub-species, of sub-buteos.” (0.09 MB DOC) [file pone.0005460.s001.doc]

| First genus in pair | Number of species in genus | Mean number of subspecies per species | Second genus in pair | Number of species in genus | Mean number of subspecies per species | Source |
| --- | --- | --- | --- | --- | --- | --- |
| *Alca* | 1 | 2 | *Pinguinus* | 1 | 1 | Baker et al. (2007) |
| *Anarhynchus* | 1 | 1 | *Peltohyas* | 1 | 1 | Baker et al. (2007) |
| *Aphriza* | 1 | 1 | *Calidris* | 19 | 2.05 | Baker et al. (2007) |
| *Attagis* | 2 | 2 | *Thinocorus* | 2 | 3 | Baker et al. (2007) |
| *Bartramia* | 1 | 1 | *Numenius* | 8 | 2 | Baker et al. (2007) |
| *Burhinus* | 7 | 2.71 | *Esacus* | 2 | 1 | Baker et al. (2007) |
| *Cerorhinca* | 1 | 1 | *Fratercula* | 3 | 1.7 | Baker et al. (2007) |
| *Cladorhynchus* | 1 | 1 | *Himantopus* | 2 | 3 | Baker et al. (2007) |
| *Creagrus* | 1 | 1 | *Rhodostethia* | 1 | 1 | Baker et al. (2007) |
| *Elseyornis* | 1 | 1 | *Thinornis* | 2 | 1 | Baker et al. (2007) |
| *Gallinago* | 16 | 1.43 | *Coenocorypha* | 2 | 3 | Baker et al. (2007) |
| *Haematopus* | 11 | 1.54 | *Ibidorhyncha* | 1 | 1 | Baker et al. (2007) |
| *Heteroscelus* | 2 | 1 | *Tringa* | 10 | 1.6 | Baker et al. (2007) |
| *Hydrophasianus* | 1 | 1 | *Jacana* | 2 | 4.5 | Baker et al. (2007) |
| *Larus* | 43 | 1.6 | *Rissa* | 2 | 1 | Baker et al. (2007) |
| *Limicola* | 1 | 2 | *Philomachus* | 1 | 1 | Baker et al. (2007) |
| *Limnodromus* | 3 | 1.66 | *Lymnocryptes* | 1 | 1 | Baker et al. (2007) |
| *Microparra* | 1 | 1 | *Irediparra* | 1 | 1 | Baker et al. (2007) |
| *Nycticryphes* | 1 | 1 | *Rostratula* | 1 | 2 | Baker et al. (2007) |
| *Pluvianellus* | 1 | 1 | *Chionis* | 2 | 2.5 | Baker et al. (2007) |
| *Stiltia* | 1 | 1 | *Glareola* | 7 | 1.7 | Baker et al. (2007) |
| *Celeus* | 11 | 4 | *Meiglyptes* | 3 | 2.66 | Benz et al. (2006) |
| *Dryocopus* | 7 | 3.93 | *Mulleripicus* | 3 | 2.33 | Benz et al. (2006) |
| *Picumnus* | 26 | 2.7 | *Sasia* | 3 | 2 | Benz et al. (2006) |
| *Reinwardtipicus* | 1 | 2 | *Chrysocolaptes* | 2 | 8.5 | Benz et al. (2006) |
| *Veniliornis* | 12 | 3.08 | *Picoides* | 12 | 4.5 | Benz et al. (2006) |
| *Gypaetus* | 1 | 3 | *Neophron* | 1 | 3 | Lerner & Mindell (2005) & Griffiths et al. (2007) |
| *Gyps* | 8 | 1.3 | *Necrosyrtes* | 1 | 1 | Lerner & Mindell (2005) & Griffiths et al. (2007) |
| *Haliastur* | 2 | 2.5 | *Milvus* | 2 | 4.5 | Lerner & Mindell (2005) & Griffiths et al. (2007) |
| *Harpia* | 1 | 1 | *Morphnus* | 1 | 1 | Lerner & Mindell (2005) & Griffiths et al. (2007) |
| *Lophoictinia* | 1 | 1 | *Hamirostra* | 1 | 1 | Lerner & Mindell (2005), Griffiths et al. (2007) & Lerner et al. (2008) |
| *Torgos* | 1 | 3 | *Aegypius* | 1 | 1 | Lerner & Mindell (2005), Griffiths et al. (2007) & Lerner et al. (2008) |
| *Limnothlypis* | 1 | 1 | *Protonotaria* | 1 | 1 | Lovette & Berminham (2002) |
| *Microligea* | 1 | 2 | *Xenoligea* | 1 | 1 | Lovette & Berminham (2002) |
| *Ampeliceps* | 1 | 1 | *Gracula* | 2 | 5.5 | Lovette & Rubenstein (2007) |
| *Dumetella* | 1 | 1 | *Ramphocinclus* | 1 | 2 | Lovette & Rubenstein (2007) |
| *Margarops* | 1 | 4 | *Cinclocerthia* | 2 | 3 | Lovette & Rubenstein (2007) |
| *Scissirostrum* | 1 | 1 | *Enodes* | 1 | 1 | Lovette & Rubenstein (2007) |
| *Speculipastor* | 1 | 1 | *Grafisia* | 1 | 1 | Lovette & Rubenstein (2007) |
| *Streptocitta* | 2 | 1.5 | *Sarcops* | 1 | 3 | Lovette & Rubenstein (2007) |
| *Conioptilon* | 1 | 1 | *Gymnoderus* | 1 | 1 | Ohlson et al. (2007) |
| *Doliornis* | 2 | 1 | *Ampelion* | 2 | 1.5 | Ohlson et al. (2007) |
| *Perissocephalus* | 1 | 1 | *Cephalopterus* | 3 | 1 | Ohlson et al. (2007) |
| *Phoenicircus* | 2 | 1 | *Rupicola* | 2 | 2.5 | Ohlson et al. (2007) |
| *Pipreola* | 11 | 2.2 | *Ampelioides* | 1 | 1 | Ohlson et al. (2007) |
| *Porphyrolaema* | 1 | 1 | *Procnias* | 4 | 1.5 | Ohlson et al. (2007) |
